# Rethinking Spaces of Leisure: How People Living with Dementia Use the Opportunities Leisure Centres Provide to Promote their Identity and Place in the World

**DOI:** 10.1007/s41978-022-00121-x

**Published:** 2022-10-27

**Authors:** Christopher Russell, Geoffery Z. Kohe, Shirley Evans, Dawn Brooker

**Affiliations:** 1 Association for Dementia Studies, University of Worcester, Henwick Grove, Worcester, WR2 6AJ UK; 2 School of Sport & Exercise Sciences, University of Kent, Chatham, UK

**Keywords:** Dementia, Identity, Serious leisure, Social citizenship, Space

## Abstract

We report on research that found joining activities within community leisure and fitness centres (Centres) enabled people living with dementia to create meaning about everyday life and foster identity. Focusing on three Centres in England, the study was informed by the experiences and accounts of four people living with dementia, their life-partner (if applicable) and the sports professional most closely associated with the person as each participated within a range of leisure opportunities. The methodology was underpinned by phenomenological philosophy and utilised participative methods. Theoretically, the paper draws upon considerations of serious leisure that provide ways in which the participants’ experiences could be understood and wider implications considered. Conceptual themes we derived from the data analysis were place, citizenship, and belonging (where the Centre acting as a physical space was important); identity and interaction (where the focus was upon space making and embodiment); safe spaces and care (i.e., how wellbeing was sustained and how participation and meaningful engagement occurred within the space); and, the value of Centres as opportunity structures (where all of these themes coalesced). Amid current public health debates over resourcing and care, this research provides timely insights and continued needed debates on the relationship between adequate social, economic and political support/resourcing, and the ability of Centres to facilitate and sustain meaningful and safe spaces. Beyond, we suggest our findings offer learning that might extend to wider contexts; for example, through including Centres within social care and health initiatives, where emphasis will be upon participation as a citizen rather than as a patient.

## Introduction

Community leisure and fitness centres (Centres) are sites situated within local neighbourhoods, which are open to the public, and provide a range of opportunities for physical activity and/or socialisation. Typically, this may include chances to take part in sport (for example badminton, table-tennis, five-a-side-football etc.), physical activity within a gymnasium, or utilise associated facilities such as a café/restaurant or communal meeting space. Provision may also include fitness classes or incorporate a swimming pool, physical rehabilitation facility, or clinic (Taylor et al., 2011). In England, the contextual focus for this article, Centres might be privately run, however the majority are provided by charitable trusts or local councils.

Contents, uses and ownership notwithstanding, Centres do not tend to be places that people may necessarily think of in relation to people living with dementia. Yet, leisure has been shown to be a means through which the identity of individuals living with dementia, and opportunity to grow and develop, can be strengthened (Dupuis et al., 2012; Russell et al., 2020). Whilst the extent to which benefits might be realised remains ambiguous (Fortune & Dupuis, 2018), for some people Centres play important roles in their lives, and often these roles go beyond just somewhere to take exercise. As is well-established (see, for example, Casey et al., 2009; Ekholm & Dahlstedt, 2019), Centres are an important resource within communities that can foster socialisation, help develop individual and collective identities, and provide people with a sense of belonging and purpose. More specific to dementia, Russell et al (2020), for example, point to the role of local Centres in the United Kingdom in providing spaces for people living with dementia to sustain and fortify personal relations, social connections and a sense of community engagement.

Whilst there is increasing understanding about how a sense of place contributes to adjustment there remain uncertainties with how specific settings within neighbourhoods might contribute (Odzakovic et al., 2020). This research thus aimed to explore whether and how the spaces within leisure settings could be used by participants to strengthen feelings of identity and ‘being in the world’ (discussed further below). Additionally, we sought to investigate the notion of Centres as spaces in which greater attention to care and support might be fostered through sustaining wellbeing, participation and meaningful engagements.

## Dementia and Identity

Dementia is a syndrome caused by an extensive range of disorders which gradually hinder the brain and cognitive performance over time, with Alzheimer’s type dementia being the most frequently reported (Russell et al., 2020). People with dementia live with a diverse and fluctuating range of changing symptoms and pathologies. These include impaired learning and recall, trouble with speech and comprehension, alterations in behaviour and diminished ability to engage in activities (Alzheimer’s Disease International, 2014). Adjusting to these symptoms is challenging, and individuals experience dementia in different ways (Brooker & Latham, 2016). Understanding of dementia has increased but pharmacological interventions to modify symptoms remain in their infancy (Alzheimer Europe, 2021). National Dementia Strategies, including the World Health Organisation Action on Dementia (World Health Organisation, 2017), emphasise the importance and significance of early diagnosis, neighbourhood and community (Chow et al., 2018; Odzakovic et al., 2021). The symptoms of dementia causing illnesses undermine comprehension people have of who they are, and have malign, corrosive outcomes for individuals and their families (Brooker & Latham, 2016; Kitwood, 1997). However, maintenance of a strong sense of identity can enable adjustment by the person to life with dementia in ways that allow for a happier, more settled future (Bunn et al., 2012; Cheston, 2013).

Subsequently, our research focused upon social identity to help investigate what went on for some individuals within Centres. Buoyed by previous research undertaken by two of the co-authors of this paper (Brooker et al., 2017; Evans et al., 2021; Evans et al., 2020), we worked from an initial position in which it felt likely that social interactions and discourses would be inherent to Centres. Specifically, that Centres were sites accessible to wide public groups, had diverse characteristics (e.g., ranging from large open areas for physical activity classes, through to smaller areas dedicated to fitness), and included alternative places, such as cafes, restaurants and meeting spaces. From this work, and congruent with scholarship on the roles of social spaces in identity formation (Spracklen, 2013), it appeared that there would be multiple opportunities for social interaction to occur that might enable individuals to both find meaning within, through and about the space and to make meaning about their identity.

As is well established, identity is fluid, changing for individuals based on their experiences and interpretations thereof (Snyder and Spreitzer, 1979). Erikson (1995) further argues that identity is built by experiences that foster opportunities to express one's individuality and receive feedback and validation from others. Because of the frequent interactions physical activity participation may produce, within the context of analysing Centres notions of how self-contextualization might happen within intergroup contexts,—where individuals define themselves, and behave as group members as well as individuals, understanding themselves as ‘we’ and ‘us’, as well as ‘me and ‘I’ (Stevens et al., 2017; Wearing, 2011) – can, therefore, be seen as important. Whilst a person’s sense of identity plays a significant role in their understanding of who they are, dementia can erode this with consequent negative impact upon well-being (Brooker & Latham, 2016; Kitwood, 1997). This is often allied to a sense of stigma felt by people living with dementia, which is linked to their diagnosis and how others may view them within the context of everyday life; for example, presuming a lack of ability to make decisions about and/or participate activities of daily life (Butchard et al., 2020). As Fletcher (2021) highlights, it is ‘felt stigma’ (e.g., negative self-appraisals on the part of people living with dementia and fears regarding the reactions of others) which is more pervasive and harmful to individuals’ sense of self and their confidence than active discriminatory behaviours by third parties. Social identity, therefore, is of specific relevance to life with dementia because of the necessity to focus upon seeing people with dementia as people, rather than simply medical cases with a collection of symptoms to be addressed (Kitwood, 1997).

As explored in this paper, understanding of oneself generated through ongoing engagements with others is a core tenet of contemporary dementia scholarship, especially as this relates to the social citizenship of people living with dementia (Barrie, 2019). The social sense of identity also matters because of the significance relationships hold for people living with dementia, and how these connections change for individuals as their symptoms develop over time (White et al., 2019; Walsh, et al., 2019). Accordingly in our study, and harmonising with its use in sport and leisure contexts (Spracklen, 2013), identity was understood in a social sense; of belonging and comprehension individuals held of where they fitted into the social structures around them. The diverse social spaces offered by Centres provided different possibilities for identity formation for individuals with dementia and adjustment to life with dementia.

## Dementia, Space and Social Citizenship

The research was located in the United Kingdom, specifically in the West Midlands and South-West of England. Within the conceptualisation of identity, we felt it prudent to acknowledge that identity formation is contingent upon historical and socio-cultural understandings prevalent within particular locales (Holliday, 2010). Thus, the geographic setting of the research meant interpretations held about identity by participants would likely to be influenced by prevailing understandings held within this ‘western’ part of the world, and individual’s distinct socio-economic, ethnic, cultural, political and geographic backgrounds. In this study participants may be predominantly characterised as white European, though with varied socio-economic backgrounds and socio-geographic experiences (see Sect. 6.1). Such recognition is important because the research involved exploration of the identity of people living with dementia, and identity traits valued within western cultures, for example autonomy/independence, are ones upon which the symptoms of dementia are liable to have adverse impact (Birt et al., 2017).

Two of the three research sites were Centres run by charitable trusts on behalf of local councils. The remaining venue was provided on behalf of a privately run organisation. It was included because the scope of the organisation, which was sufficiently large to offer facilities right across England, meant that the range of opportunities to participate in physical activity and the nature of the space available matched those offered by conventional Centres provided on behalf of local councils. Consequently, there exists variability across Centres in this study in terms of the physical and human resourcing, facility and activity provision, and ways social space is created and sustained.

Congruent with earlier noted scholarship on the value of community resource provision, we have acknowledged the ability of Centres to offer a variety of spaces, large, small, indoor, outdoor, as relevant to matters relating to social identity. The fact these spaces tend to be located within neighbourhoods close to where individuals live was also notable (Odzakovic et al., 2021; Ping Kung & Taylor, 2010). Spaces situated within neighbourhoods enable everyday citizenship to be manifested by people with dementia through social relations, experiences and practices (Nedlund et al., 2019). In terms of relationships, emphasis is placed on norms, practices, meanings and identities (for example, how individuals perceive themselves in the context of their day-to-day interactions with others) and social interaction within the everyday spaces of life (Barrie, 2019). Offering opportunity for people with dementia to sustain their sense of being in the world, Centres provide physical activity engagement, and spaces that surround those activities, in which individuals may position themselves within, and contribute to, a distinct community space. There exist within Centres valuable opportunities to participate in routine interactions (e.g., locker room and pre-exercise conversations, convivial banter, purchasing food and drink, sourcing equipment etc.) that also define the space.

Conceptualisations of identity notwithstanding, it is essential to ensure that the heterogeneity of people living with dementia is recognised (Thomas & Milligan, 2018). Furthermore, as we explore below, there are challenges to consider. For example, related to how well expressions of individuality are enabled within the spaces and how far a sense of belonging among participants can be fostered. Moreover, consideration must be afforded to inclusivity and characteristics of difference that will influence the identity of people with dementia, as part of membership of the societies within which they live (Bartlett et al., 2018; La Fontaine et al., 2007; Villar et al., 2019).

## Dementia, Space and Leisure

Social interaction within the everyday spaces of life can be understood and examined within the frameworks offered by theories of leisure, particularly wherein individuals are able to make choices about engagement in activity, and its nature. Leisure can be categorised as casual, offering immediate, inherently rewarding but short-term pleasure for which no training or expertise is required (Stebbins, 1997). Yet, additionally, leisure activities can offer more than the fulfilment of tasks, potentially offering individuals interest and meaning (Spracklen, 2013; Torkildsen, 1999). The theoretical understanding of serious leisure (Cohen-Gewerc & Stebbins, 2013; Stebbins, 1992), explains leisure as the regular progression of amateur activities that are substantial, of interest and fulfilling, and where skills, knowledge, and experience are acquired and enacted (Stebbins, 1992). Extending understandings of social identity, citizenship and dementia, we considered that Centre spaces, and activity offered therein, might contribute to understanding how participants could demonstrate agency and thus their social citizenship, potentially through the enactment of serious leisure. Serious leisure, scholars note (Stuart, 2022), can be used by individuals to support transitions in life/through the life course. As such, given that successful adjustment to life with dementia is achieved through a process of transition, it felt pertinent to focus on how participants living with dementia used leisure to reframe their own sense of self.

Our work also aligns with the broader arguments regarding the importance and intersections between the holistic, physical, social, economic and political aspects of space. Most significantly, our research is in synergy with the breadth of scholarship that has firmly established the role of leisure, sport, and physical activity spaces on individuals’ bodies, experiences and interactions. These considerations have been underpinned by the work of sport and spatial theory scholars who have offered insight into interactions between participants and use of their bodies within spaces as they engaged in physical activity (e.g., Bale, 1996; Newman & Falcous, 2012; Olson, 2017; van Ingen, 2003; van Ingen et al., 2018). Such conceptualisation aligned with the phenomenological underpinnings of our methodology, and comprehension drawn from this philosophy relating to embodiment and ‘being in the world’ offered the means through which understanding could be garnered. The focus here is upon the use of space as concept conveying the complexity of a site’s and body’s social, political, cultural, economic dimensions.

## Method

### Methodology and Design

The sites of the research were three Centres, all located in the Midlands or South West of England. The choice of Centres was directly related to the involvement within each of the research participant living with dementia. Each Centre was located within a different town, each varied in size, the smallest having a population of approximately twenty-thousand people, the largest a population around one hundred thousand. There were eleven research participants included within the study. Four people living with dementia were recruited as primary research participants. Three of these individuals attended along with their spouse, and each of those three were also included as participants, alongside four Centre Workers who respectively had closest involvement with each participant living with dementia. Additional details are set out in Sect. 6.1 (below) where short biographies of every primary participant are included. Data collection took place between October 2017 and November 2018.

Recruitment criteria were relatively few and widely drawn: i.e. a person living with dementia, regularly engaging in sport/ physical activity at a Centre, who held mental capacity (Department for Constitutional Affairs, 2007) to participate in the research. Criteria were designed in this way, in part, because of presumed challenges recruiting people living with dementia who were engaging at Centres. Study of the literature indicated that whilst people might engage in leisure activity in spaces inhabited by the public (Dupuis, et al., 2012), participation in vigorous physical activity in the context we planned to investigate was rarer (Atherton, et al., 2016). Early in the recruitment stage an extensive approach to maximise opportunity of gathering participants living with dementia was initiated. For example, via enrolment of the lead researcher in the ‘Join Dementia Research’ programme run by the Alzheimer’s Society, and through advice from the Dementia Engagement and Empowerment Project (DEEP) – the charity that promotes and coordinates initiatives led by people with dementia in the United Kingdom. An earlier scoping phase of the project had employed a questionnaire, sent to Centres across England, to ascertain the nature and level of participation of people living with dementia in physical activity within the sorts of venues we were interested in. Responses enabled the lead researcher to be in contact with Gatekeepers who made contact details available to people who might have an interest in being recruited. This was congruent with underpinning ethical research principles requiring autonomy for, and nonmaleficence towards, participants at every stage (Artal & Rubenfeld, 2017; Sobočan et al., 2019). Three of the four primary participants were recruited in this manner, and elected that the employee at their Centre who knew them best make contact on their behalf. One participant, Ivan (see below), was recruited having heard about the research through being a member of his local Dementia support group. He made contact through the facilitator of that group. With the informed consent of primary participants, the family carer of each person, and Centre Worker most closely involved with their engagement at the Centre, were then also engaged as research participants. As outlined later, one participant, Jacqui, lived alone, and was estranged from her family, thus no family carer was recruited.

The decision to recruit four research participants living with dementia was motivated by the wish to gather accounts that were as rich and comprehensive as possible. This size of sample afforded opportunity to collect in-depth, meaningful, nuanced data, borne out in several interviews and observations over a period of more than twelve months, the richness of the data shedding light upon an under-researched area (Smith, 2017). Additionally, the potentially small general cohorts of accessible participants living with dementia, engaging in the activity of Centres, influenced decisions, and meant the project was set toward a small sample by nature. However, the scope extended beyond this because family carers and Centre Workers were also recruited to help supply valuable contextual detail.

The social citizenship focus informed the research methodology and linked to the importance and relevance of employing a phenomenological approach to understand what ‘being in the world’ and what one’s place within that meant for participants (Nedlund et al., 2019). Subsequently, Heidegger’s (1962) interpretive phenomenology was particularly of value. Heidegger argued that humans are not held to the present but have a consistent sense of projecting themselves towards the future. He characterised this as ‘authentic temporality’ (Heidegger, 1962). The associated notion of temporality provided additional utility because dementia illnesses are progressive, for example people may experience difficulties with memory, especially short-term recall, over time. Thus, the passage of time, and its meanings to people were of relevance. In addition, people living with dementia are likely to be aware of the developing nature of dementia causing diseases and feel the consequences of this deeply (Oliver, 2016; Swaffer, 2016). Consideration of authentic temporality yielded numerous methodological benefits especially withing the data collection process. For example, the year-long data collection period enabled time for relationships to be built between researcher and primary participants and feel at ease discussing matters potentially liable to cause anxiety (e.g., discussions about the progressive nature of symptoms and worries about short term memory loss).

Additionally, the methods were informed by the phenomenology of empathy, articulated by Edith Stein (2000), and contributed primarily to how the researcher engaged alongside participants to enable each person to feel able to articulate their feelings and opinions. Stein believed that empathy is fundamental to discovering truth (Sullivan, 2002), and her phenomenology counselled understanding of oneself as a holistic, experiential entity, whilst seeking insight from others. This is dependent upon feeling and demonstrating concern for a person, and endeavouring to understand what experiences might mean for them (Stein, 2000; Svenaeus, 2016). Building upon this, our enquiry was also supported by understanding of phenomenological approaches addressing embodiment and embodied consciousness. Specifically, a phenomenological approach aided our comprehension of how participants used their bodies as a form of meaning making (as we evidenced, through observation of physical activity engagement, and non-verbal and para-verbal communication). As phenomenology pioneer Merleau-Ponty (1962) argued, the body has its own sense of agency, and its actions can express meaning in relation to identity, the phenomenon known as ‘embodiment’. As Merleau-Ponty (1962), and others (Hockey & Collinson, 2007; Kissow, 2015), contend, conceptualisations of embodiment are of value in assisting exploration of the role and meaning of the physical space within the shaping of identity for participants. Our deployment of phenomenology here echoes scholars who have illustrated ways individuals with cognitive disabilities use familiar environments to negotiate identity (Phinney et al., 2007). Within the Centre context, our specific attention is on the significance of previous experiences participants hold of space and activity, their immediate perception of the experience (Lundberg et al., 2011), and how spaces are affectively sensed (Bakewell, 2017).

Conceptually, we appreciate the valuable links here to notions of ‘sensuous’ geography (Rodaway, 2002); where people create meaning within familiar spaces through use of their senses. In sporting contexts scholars have illustrated this in analyses of ways specific venues influence how participants express themselves physically. For example, using understanding of the terrain to determine how they balance and position themselves whilst engaging in displays of physical prowess (Hockey & Collinson, 2007). That Centres were likely to be familiar to participants, because of their regular involvement within them, therefore made such an approach even more useful—because of how individuals can create meaning within familiar spaces using their senses.

The methodology required involvement by the lead researcher in activities with and alongside participants within the Centres. While necessary for data collection, it also aided methodology rigor because of the researcher’s positionality. Specifically, their previous experience in professional social work involving people living with dementia and their families aided the building of trust, rapport and empathy with participants, and tailoring interviews and observations to the needs of individuals. There remained, however, the possibility this presence might influence participants' behaviours. Thus, reflexive approaches were employed to address any inadvertent and unwanted outcomes (Finlay, 2002). This included use of a research diary to monitor and understand reflective interventions and techniques (Etherington, 2004) in which details of every research contact were noted alongside initial reflections, which were reviewed before future interviews or observations were convened. In addition, there were regular and frequent discussions between members of the research team, collectively and separately, throughout the research (e.g., in relation to refining interview and data collection techniques, interview content and ethical issues, emerging ideas and conceptual links).

### Methods

Ethical approval for the study was gained from the Health and Science Research Ethics Committee at the University where the lead researcher was based.

To aid data collection, traditional methods of enquiry, such as the long interview—where researcher and participant meet, and where issues of relevance to the research question are appraised (McCracken, 1988)—were initially included. However, such methods could not entirely encapsulate understanding of individual’s meanings of moments, and the methodology was revised to use go-along interviews; whereby the researcher accompanied and took part in certain activities that were the subject of data collection, whilst contemporaneously seeking participant accounts (Odzakovic et al., 2021). Aligning with focus on embodiment (Kusenbach, 2003), specific participant observations were selected to enable understanding of how participants utilised sporting endeavour and their chosen physical activity spaces to help shape their identity and place in the world. Go-along interviews provided opportunities for participants to articulate the immediate experience, and recall with more ease their previous experiences, through repetition of physical activity. The approach was consistent with other dementia studies that highlight how methods must anticipate the likelihood that the symptoms of dementia causing illnesses may erode the ability of some participants to use speech to express themselves (e.g., Genoe & Dupuis, 2011; Wright, 2018). Informed consent to participate in the study was obtained from participants as part of standard university ethical approval processes. Data was analysed inductively whereby the lead author, in conjunction with the research team, undertook a process of thematic coding, organisation and evaluation; using intra-coder reliability and intercoder consistency and confirmability checks to develop a consensus on the conceptual coding framework and data interpretation (Braun & Clarke, 2006, 2013; O’Connor & Joffe, 2020). Further trustworthiness was also gained through member-checking transcripts for veracity, accuracy and intention. The coding framework was utilised to analyse individual participant experiences further, with subsequent clarifications made to assumed meanings of phrases and participant’s intended expressions. Analysis was then undertaken of the entire participant group to identify points of congruity, difference, and/or interest within the narratives. Cross-participant analysis also enabled deeper consideration of the prevalence and relevance of incidents, moments and meanings within experiences, and contributed to the wider project critique about the overall place of sport, physical activity and Centres within participants’ lives. This approach was consistent with our epistemological and paradigmatic positions as qualitative researchers, and advantageous in affording a way in which the insights and experiences of participants could be scrutinised, considered, and understood.

## Findings

### Research Participant Biographies

Short biographies of the four participants living with dementia help elucidate findings. Each person is given a pseudonym to afford anonymity in line with ethical considerations.

Ivan was sixty-four years old at the time of the research. He had lived his life in the West Midlands of England and physical activity was important to him, for example, he had always regularly played sport and engaged in physical activity. In earlier years that had included cricket and football. Latterly he was a member at his local Centre where he took part, often alongside his wife Jemma, in fitness classes and gym sessions, and had membership there for approximately five years prior to diagnosis. His lifelong love of sport and physical activity lay behind his motivation for membership. These were traits shared by Jemma. Ivan had been diagnosed at the age of fifty-seven with corticobasal degeneration, a rare form of dementia causing illness, which resulted in increasingly severe difficulties with speech, and with challenges to movement and dexterity. Ivan remained able to remember events with relative clarity but really struggled to articulate them verbally. Kyle, the Centre Worker with whom Ivan often took part in fitness sessions within the gym, was included as a research participant.

Paul was seventy-nine years of age and lived in the south-west of England. Sport had played a fundamental part in his life, for example he had been a skilled cricketer and footballer, playing regularly for his local amateur teams. Later Paul had taken up administrative roles with these clubs. He now participated in leisure activities alongside his wife Connie at the Centre close to where he lived. Connie, who enjoyed sport and physical activity but to nothing like the same extent, affectionally and with humour described herself as a “sporting widow”. Paul had been diagnosed with Alzheimer’s Disease two years before the research began. Symptoms included short-term memory loss, and anxiety. He had lost confidence in being able to follow his train of thought, and this impeded his willingness to speak in public. Paul had difficulty remembering details about recent events but retained the ability to recall things that happened longer ago. Paul and Connie had begun using their local Centre after his diagnosis, having heard about a group there with a specific offering for people living with dementia and family carers. This was facilitated by Martin and Jane, Centre Workers. Paul had got to know them well. This, and the frequent interaction he had with both as he took part in physical activity at the Centre, meant Martin and Jane were included as research participants also.

Leonard was aged fifty-nine at the time of the research. Historically he had disliked sport and only came to it later in life, in the years after his diagnosis, regularly attending alongside his wife Caroline, at the same Centre as Paul and Connie, which was approximately ten miles from where the couple lived in the South-West of England. Leonard enjoyed playing badminton and swimming there. As with Paul and Connie, Leonard and Caroline were well acquainted with Centre Workers Martin and Jane. Leonard valued the time both took to get to know him and provide opportunities to engage in physical activity which met his needs and aspirations. He had been diagnosed with Posterior Cortical Atrophy three years before the research began. This is a form of dementia causing illness resulting in compromised visual ability and spatial judgement, as well as having impact upon short-term memory. Leonard had little difficulty with speech, although occasionally felt muddled, struggling to find words as swiftly as he would like.

Jacqui was sixty-three years old and resided close to the town where she had been born and raised in the West Midlands of England. Physical activity had been of fluctuating importance to her throughout her life. However, since she had retired on grounds of ill health caused by dementia Jacqui had attended her local Centre regularly and frequently. She enjoyed exercise classes and walks organised by staff at the Centre in particular. She was close with Patrick, the Centre Worker who held responsibility for organising the activities Jacqui most enjoyed and in which she took part most frequently. Patrick was therefore included as a research participant. Jacqui was diagnosed with dementia with Lewy bodies. She had difficulty with posture and physical dexterity, alongside compromised ability to read and write. Jacqui also had minor hallucinations which hindered her ability to orientate herself to her surroundings and caused some short-term memory loss. Living alone, Jacqui attended the Centre solo.

### The Centre as a Space for Leisure, Identity Formation and Social Citizenship

This section describes how spaces influenced identity formation and contributed to the strengthening of social citizenship for participants living with dementia. The broad themes from the research were place, citizenship, and belonging (where the Centre acting as a physical space was important); identity and interaction (where the focus was upon space making and embodiment); safe spaces and care (how wellbeing was sustained, and how participation and meaningful engagement occurred within the space); and the value of Centres as opportunity structures (where all of these themes coalesced).

To assist understanding and to elucidate arguments we contextualise the engagement of participants within two distinct, but linked/ over-lapping milieux, which we refer to as ‘realms of everyday life’:

1. Space Making
2. Using time within the space: the individual and their interactions (relationship crafting)

Space making incorporates place, citizenship, and belonging, with the Centre acting as the physical space within which these occurred. It therefore concerns the location and nature of the environment and meaning in relation to the identity and social citizenship of participants. For example, spaces offered venues for participants to use their bodies to express meaning through physical ability and prowess. The second realm, using time within the space, involves how the space was used and by whom; specifically, how individuals were able/or inhibited in expressing meaning about their identity and place in the world. This included recognition of the identity participants held and how engagement in activity and interaction with others in the spaces influenced this. It also concerned how time was used to promote safe spaces and care, for example through sustaining wellbeing, participation and meaningful engagements within the space.

#### Space Making

Here we explore the physical nature of Centres and their meaning in relation to the identity and social citizenship of participants. Whilst it was the spaces themselves that were the primary focus of enquiry, the location of Centres also played a part identity construction, and thus brief mention is warranted. For example, as the biographies (above) show, Centres were located in neighbourhoods familiar to participants. This offered scope for each person to do what is described in the literature and, “claim their place in the community” (Phinney, et al., 2016 p.389) through engaging with the offering at a location they were well acquainted with. For example, Leonard’s reflections on this:

“…it’s being used to the place that you’re in…isn’t it. Used to being here.”

Leonard interview at his Centre, 2nd July 2018.

Additionally, Jacqui’s keen attachment to the geographic space and place of her birth and upbringing, her affinity for the town:

“It’s just born and bred; it’s just how you feel”.

Jacqui: interview at her Centre, 4th October 2018.

Enabling such ongoing expressions of individuality, often alongside others within their neighbourhood was especially valuable because, as scholars have argued, it is within such ordinary places that social citizenship is fostered (Bartlett & Brannelly, 2019; Bartlett, 2016). However, it was the physical spaces themselves that offered venues for participants’ to use their bodily expression and meaning-making. For example, as Leonard reflected:

“…cos’ a large swimming pool, you can do, you know it’s big enough to do what you want to do… I can throw balls from one end of the swimming pool to the other end of the swimming pool”.

Leonard interview at his Centre, 2nd July 2018.

Elements of Rodaway’s (2002) sensuous geography at play were also evidenced in individuals’ ability to use their senses to create meaning within these familiar spaces. Thus, for example, Leonard related:

“It takes your breath away. It’s brilliant.”

Leonard interview at his Centre, 3rd May 2018.

Here, and similar to the ways described by Hockey and Collinson (2007), it was the immediate perception of the experience through use of one’s senses that was significant. This occurred within the physical spaces of the Centres. These were spaces designed to enable varied sporting activity, providing opportunities for participants to use their bodies to express meaning about their identity and place in the world. Embodiment is significant, because how one uses one’s body to engage in physical activity is an expression of meaning (Merleau-Ponty, 1962). Thus, Ivan engaged vigorously and purposefully in the gym, demonstrating aspects of his identity such as resilience.

“…when undertaking the exercises Ivan’s face was set in an expression of extreme concentration. He was taking what he did very seriously.”

Ivan: observation at his Centre, 16th December 2017.

This example, taken with another described later, are all instances where Ivan uses participation in physical activity to frame himself *as* a person of resilience; suggesting, a feeling he may have of a strong(er) dimension of his identity rather than being *just* a character trait. In addition, for Ivan, it appeared that physical activity felt substantial, interesting, and fulfilling, in the manner Stebbins (1992) described serious leisure. For example, as Kyle, the Worker who supported Ivan’s participation at the Centre, reflected about his approach.

“Hard working. You can see he’s trying… It’s just how he is as a person… You can see that… And you can also see that when he’s not doing it right he gets very frustrated. Very frustrated… It’s determination to do it right.”

Interview with Kyle at the Centre, 10th April 2018.

In the sort of ways described by Cohen-Gewerc and Stebbins (2013), he could also continue to hone related skills which was affirming and rewarding, providing him with feelings of self-development, self-expression, and accomplishment. As Ivan’s wife, Jemma, related.

“He likes to do the classes … he looks forward to actually going through the exercises and feeling he’s doing as much as he can… He really struggles… (but) I think he feels he’s achieved something.”

Interview with Jemma at her home, 20th March 2018.

This supported Ivan to work towards successful readjustment to dementia, as suggested happens for individuals by Bunn et al (2012) and Cheston (2013). He could continue with his life-long love of physical activity, and by engaging so whole-heartedly, express agency (Genoe, 2010) and make personal meaning (Habermas, 1990; Spracklen, 2013). This was one example of how the Centre was valuable as a site through which serious leisure could be enacted, and also legitimised and sustained within the experiences of participants living with Dementia. It also illustrated the importance of social connectivity/networks of the sort described by Wiersma and Denton (2016) and Rodriguez et al. (2018) (detailed below).

From the capacities of physical activity to be interactive and reciprocal, there exists possibilities understanding its social potential (Kontos et al., 2017; Van Manen, 1997). Thus, Leonard’s expressive approach to his participation in the space offered him opportunity for sociability he would not, necessarily, have had without his engagement at the Centre. For example:

‘Leonard smiled or laughed throughout the games. We both played hard and there was lots of heavy breathing and sweat on both sides’.

Leonard: go-along interview at his Centre, 22nd January 2018.

To recall, Ivan had been diagnosed with corticobasal degeneration, with that illness’ consequent impairment of speech. Accordingly, Ivan utilised paraverbal communication with fellow gym users to manifest his sociability and seek confirmation of his authenticity and legitimacy as an individual exercising within that physical space. For example:

‘…(an)other gym user greeted Ivan warmly as they passed with a big smile. Ivan nodded to her in recognition.’

Ivan, observation at his Centre, 16th December 2017.

Thus, congruent with Hughes’ (2019) considerations of how one constructs their sense of authentic self, the physical nature of Centres prompted and fostered meaning in relation to how participants understood their identity and social connections as they engaged in leisure. These sorts of spaces, and how they are created to cater for members, were considered important in how these individuals living with dementia made sense of the world (at a time when this is increasingly becoming difficult due to their condition), connected to others (if they wished), or be the person they wanted to be (through participating in activities or interactions) within the space. For example, as explained earlier, the size, scope, nature and location of Centres (e.g., often conveniently situated within familiar territory and neighbourhoods to where individuals lived) mattered. Each Centre provided amiable conditions for space making, and enabled individuals to forge and consolidate identities through the positive social interactions (e.g., as in examples offered by Leonard and Ivan). There were, however, challenges relating to these processes. These included being contingent upon the level of individual engagement, and how social interaction operated. We turn to these matters next. Broader challenges were also in play, relating to Centre resourcing and locations of the Centre, for example. Whilst beyond the immediate scope of this article, they are inherent to its context and must be considered in inquiries that follow.

#### Using Time within the Space

It was not solely the nature of the leisure spaces that were used by participants that enabled the maintenance of identity and sustenance of social citizenship. How individuals utilised the time spent within those spaces was also important in expressing meaning about their identity and place in the world. This further mattered because of the biographical discontinuity caused by dementia for individuals (Williams, 1994). Also, because interests and experiences people hold must form part of their ongoing identity (Brooker & Latham, 2016), and the importance of opportunity for personal growth (Bartlett & O’Connor, 2010).

Centres could offer a “place of belonging” (Phinney, et al., 2016, p.387) where individuals could feel their authentic selves, in the manner described by Tregaskis (2003). Yet, much depended on what individuals brought in terms of life history and sense of themselves. Embodied actions were thus significant. As Wright (2018) argues, people living with dementia are able to use embodied skills to support their identity. Birt et al., (2017) suggest people continue to maintain their identity and illustrate it to those around them by how they behave. An example being Paul who used his body to express the sort of distinctiveness described by Kontos et al. (2017) in relation to his sense of self as he participated within his Centre. Paul had enjoyed playing sport all his life, as articulated in his biography (above). This remained the case, demonstrated by this example:

‘Paul and I played table-tennis for approximately forty minutes… Paul smiled… chasing shots that were at the very extent of his reach…’

Paul: participant observation at his Centre, 23^rd^ April 2018.

In this way Paul was able to affirm the authenticity of his identity (i.e. being true to his sense of self) through how he spent time within the Centre. As Carter (2016) says happens, feeling real through play. It was Paul’s choice to play table tennis. Selecting this from the numerous activities on offer, as his wife Connie related,

“He enjoys the table tennis.”

Interview with Connie at her home on 14^th^ March 2018.

Time spent engaging in leisure within Centres could also be used by participants who, unlike Paul, had no such lifelong love of sport, however. This was clearest in the example of Leonard. Until the point of his diagnosis with dementia he held little love for such activity,

“When I was at school I never played any sport…I had no interest.”

Leonard: interview at his home 19^th^ December 2017.

In adulthood Leonard had drawn upon his vocational skills and employment to help frame his identity and place in the world to a significant extent pre diagnosis. For example, as his wife Caroline confirmed, he’d held high status in an earlier role as a French Polisher, describing him as:

“…the ‘go to’ man. He was the one.”

Caroline: interview at home, 14^th^ March 2018.

Later in life Leonard had been employed as a school caretaker, reflecting:

“I just loved the job”.

Leonard: interview at home, 19^th^ December 2017.

Denied the opportunity by dementia to continue to utilise his vocational skills or draw upon the meaning, purpose and enjoyment he found in employment, Leonard turned instead to using his time within the Centre to sustain his sense of authenticity, identity and place in the world through what he did there. He expressed this through use of vigorous leisure, how he used his body as he participated being something he especially valued. For example:

“I’m not so keen on table-tennis. Cos, you can’t really whack it can you? (Leonard laughs heartily)…”

Leonard: interview at his Centre, 2nd July 2018.

The vigorous nature of Leonard’s participation being further evidenced by what he said he enjoyed about his use of the swimming pool, see page 17 (above).

In more nuanced moments Leonard articulated what he gained from engagement in leisure within the spaces of the Centres:

“…you’re achieving something aren’t you…actually doing something physically.”

Leonard: interview at his Centre, 22nd January 2018.

Leonard’s account illustrates how through physical activity participants were able to make personal progress. Thus, activity within those spaces inclined individuals towards an understanding of present and future identity in the manner described by Mayoh and Jones (2015).

Notable here is how such feelings expressed about authenticity link to safe spaces, and how they influence feelings individuals have about themselves. Safe spaces are environments characterised by respect as well as safety (Flensner and Von der Lippe, 2019). Scholarship by Robertson et al. (2020) found that public spaces can be used by walking groups involving people living with dementia to provide a safe space. This enables participants to walk with autonomy and affirms feelings of capacity and agency. Jacqui reflected this in her accounts of experiences taking part in the regular walking groups facilitated by her Centre throughout the year and based in the surrounding neighbourhood. For example:

“The weather doesn’t put me off…we just go with it…we’ve been and got soaked! It’s not too pleasant but you tell yourself it’s done you good and it’s only water….if you go on the machines it’s too easy to cheat, on a walk you can’t.”

Jacqui: interview at her Centre, 13^th^ June 2018.

Also, in the manner suggested by Bullock et al. (2017) the spaces provided by Centres could alleviate feelings of surveillance. Jacqui alluded to this when reflecting on her experiences on the walks. For example:

“I wander along and chat to whoever...I don’t know them beyond the walk.”

Jacqui: interview at her Centre, 13th June 2018.

However, this was a phenomenon she also reported when using the indoor spaces offered by her Centre. The following example concerned the availability of a large indoor space, put at the disposal of herself and her acquaintances at a regular time each week, where activity ranging from badminton to darts was on offer.

“You can get together and talk…You wouldn’t do that in a gym…you’re not getting any interaction…

It is relaxed. You can come and go. You don’t feel threatened…”.

Jacqui: interview at her Centre, 13th June 2018.

Thus, the varied spaces on offer by Centres enabled a range of ways in which participants living with dementia could affirm their identity and sustain their place in the world. Evidencing that, as conjectured earlier, the range of social spaces offered by Centres offered potential for adjustment, highlighting the importance of social identity and social networks.

Here, also, those places were perceived to act as a safe space, providing Jacqui with especially positive outcomes with regards to her social citizenship. This was poignant in light of the harm wrought by the symptoms of the dementia with Lewy bodies. Safe spaces and participant accounts can offer more than comfort, familiarity, confidence and assurance. What Jacqui articulates is akin to well-being fostered by ‘spatial dwelling-mobility’ (Mayoh & Jones, 2015, p.243). That is, well-being achieved through engagement within familiar and comforting environments, alongside opportunities for experiences presented via sporting activity. The sense of well-being, fostered in such a manner, can influence the individual’s feelings about identity. As in Jacqui’s account, this is expressed in terms of a more fulfilled sense of social identity fostered within a familiar environment.

A further instance is offered by this account taken from researcher field notes of an occasion where Paul was playing table tennis:

‘Paul smiled for most of the time…He appeared to be enjoying himself a great deal’.

Paul: reflection upon observation and go-along interview, 8th January 2018.

The effect Alzheimer’s Disease had upon his short-term memory was to corrode Paul’s confidence to engage in conversation. However, the body can express distinctiveness (Kontos et al., 2017), and Paul was employing his body to reflect what participating within his Centre meant in relation to his identity. Paul, and others who experience dementia in ways that make verbal communication challenging, can enjoy and express ‘spatial dwelling-mobility’ (see above for explanation) without need to articulate it.

Of note too is how the complexity of the role offered by Centres presented participants living with dementia with opportunity not only to make the most of safe spaces and the chance of well-being, but also for serious leisure. Jacqui verbalising, in the example above, both how serious leisure was enacted and legitimised and sustained through her experiences of achievement. Participant experiences demonstrated how engagement within the Centre allowed each to express her/his individuality in the manner wished. The value of this was enhanced because social citizenship happens in ordinary places (Bartlett, 2016), and as we have seen Centres tend to be located in such settings. Advantageously, participants were also able to take the physical activity identity beyond the context of the Centre itself (Son et al., 2009). Ivan, for example, used his experiences of leisure and physical activity at the Centre post diagnosis to shape an identity characterised (particularly by others, yet also himself) by resilience. This was shown by the following account of an observation made as one fitness session at his gym concluded:

‘Ivan’s sense of determination, and desire not to be patronised came through... (as) we walked back… It was cold and showery. I had offered to bring the car round. Ivan was clearly against this. Ivan strode purposefully ahead…the desire to carry on is a strong element of his engagement.’

Ivan: observation at his Centre, 2nd February 2018.

The poignancy of this is magnified by the fact that within three months Ivan had died as a direct result of his dementia. The role of leisure though in his identity and social citizenship appeared strong, enabling Ivan to foster and demonstrate resilience not only in the physical activities he had completed on that day but by enabling his resilience to be taken out into everyday life. In Ivan’s case this was so powerful as he was able to sustain it so close to the moment of his passing.

However, it was not solely what individuals brought to the spaces themselves that mattered. The social nature of the Centres, and identity construction and sustenance of one’s place in the world, was also enhanced through the relationships crafted therein. Earlier we outlined the significant priority given to social interaction sustaining the sense of individual identity in dementia (Brooker & Latham, 2016; Kitwood, 1997), and there is congruence with leisure and sports scholarship that illustrates that being alongside others can extend individual identity beyond the physical activity context into wider social identities (Kissow, 2015). Leonard demonstrated how the social spaces of Centres were well positioned to enable participants to experience this.

“It’s about playing together, to achieve something…not necessarily…beating the person. Winning isn’t necessary. It’s about having a good game...enjoying playing together.”

Leonard: go-along interview at his Centre, 3rd May 2018.

He related how this had changed ways in which he related to other people.

“I never used to talk to people straight away, people used to have to talk to me first. But I do that now.”

Leonard: interview at his Centre, 3rd May 2018.

These findings contribute to widening understandings of dementia and social care in health settings in two ways. First, such experiences highlight some of the ways in which individual’s physicality, experiences of daily life, comprehension of their conditions (and its effects) and social interactions are not only entwined but are, also, of fundamental importance to constructions of identity and affirmations of self-worth and belonging. As described in participants’ narratives above, the affable social conditions afforded by the Centres provide a context for physical activity *and* inscribe that activity with meanings and values that may affect positive health outcomes beyond the setting itself. In revealing the saliency of Centres as vectors of socialisation and identity making for people living with dementia, the insights extend on research that has noted the varied benefits that personal/social networks have in dementia care and broader health spaces (e.g., Cornwell & Laumann, 2015; Ellward Van Tilburg and Aartsen, 2015; Fratiglioni et al., 2000; Huxhold, et al., 2013; Wiersma & Denton, 2016). Moreover, it has been noted that the success of the provision of interventions to improve health and wellbeing via social prescription is also thought to depend in part upon engaging alongside others and realising personal assets (Payne et al., 2020).

Secondly, participants’ experiences described above also further underscore the widespread significance of building social networks both personally and collectively to enhance individual health care provision, community support structures and regional/national infrastructure and resource provision. There are no guarantees that social network formation can be created, sustained, or resourced effectively via sites such as a Centre, health care professionals, communities or the state ad infinitum (as perennial public health and social care changes and/or funding cuts in the UK and further afield frequently attest). Yet, irrespective of context or prevailing conditions, there is widespread acknowledgement that if amiable conditions for social networks are present (e.g., appropriate physical and psychological resources, provision of safe spaces, sustained and meaningful interactions, support structures, individual engagement etc.), then social networks may yield positive health outcomes (Ellwardt et al., 2015; Marseglia et al., 2019; Saito et al., 2018).

The importance of social networks, we recognise, is also part of the underlying acknowledgement within social welfare and public health research of the value of holistic approaches to care within the sector and, relatedly, wider conceptualisations of care/care ethics to underpin resourcing and provision across the board (Haggerty & Shapiro, 2013; Reamer & Reamer, 2018; Stanhope et al., 2015). In creating enticing, safe, supportive and engaging environments in which meaningful social interactions and citizenship can flourish, and the lives of people with dementia are invariably enhanced, Centres configure themselves (intentionally or otherwise) as both a structure and provider of social, health and wider community care. The data presented above also help affirm the place and roles of the Centre as a site of needed focus within dementia research; particularly in regards how such spaces are significant for individuals, but like other social care setting (e.g., hospitals, education facilities, reminiscence groups, respite centres, dementia cafes, informal groups), comprise an invaluable part of the state, local and community health and social care landscape (Baldwin & Greason, 2016; McGovern, 2015; Parker & Penhale, 2018; Steward, 2021; Tanner, 2013).

Yet, congruent with progressive challenges to make such settings more person-centred, there is also the need to ensure such spaces respect and enact not just professional obligations of care (as enshrined in law), but embody more sensitive, sensible, empathetic and socially just care ethics. There are, we note, encouraging signs in various areas of sport, leisure and health where organisations are demonstrating greater attention to members’ care and welfare. As Rietkerk et al. (2021) note, one approach is for providers to work more closely on developing bespoke plans which offer members greater agency over their health and physical activity choices. Similarly, within youth sport settings, others have evidenced benefits to participant’s experiences and overall quality of life satisfaction where person-centred approaches are adopted (e.g., Bean et al., 2020). More detailed interrogations of what this care ethics might ‘look like’ within Centres are beyond the scope of this paper. Nonetheless, we note that an appreciation of the social nature of physical activity within network formation by people living with can (and in the case of the participants above, does) form a substantive part of this consideration.

The potential for Centres to offer people living with dementia opportunity to utilise the spaces to maintain identity and sustain their place in the world through relationship crafting was enhanced by the diverse nature of those environments. In accord with dementia scholarship highlighting the value of agency in enabling people to exercise power in everyday life (Bartlett and O’Connor, 2007; Nedlund et al., 2019), we stress that flexibility of Centre spaces is important for people living with dementia because they offer opportunity for individuals to demonstrate agency in how they use them. Often, for example, rooms within Centres were of sufficient size and had the flexibility to be used for a range of purposes, including the large room in the Centre Paul used for playing table tennis. This could then double as the space for breaks from the activity and the opportunity to sit with refreshments in company. The Centre Worker closest to Paul recognised how he made use of the space when he wished to be with others:

“…just being…being with Connie…and talking to her, talking to people, looking happy…”

Martin: interview at the Centre, 3rd May 2018.

This was notable because symptoms of the Alzheimer’s disease had robbed Paul of the confidence to engage proactively and freely in discourse. The space felt a safe one where Paul could wait, watch and engage with others on terms he was comfortable with.

Feeling part of a social group can mitigate biographical discontinuity of the sort caused by dementia, through providing individuals with a sense of purpose (Anderson & Whitfield, 2013). In Paul’s case he retained his lifelong love of sport through the access the Centre gave him to his favoured leisure activities, whilst the space also enabled him to gather with and continue to enjoy the company of people in similar circumstances. As Connie said:

“We’ve been going over eighteen months …it’s like people...you’re not made to feel embarrassed or anything like that…he just enjoys going up. And he used to love being with people, he was a very sociable person”.

Connie: interview at home, 14th March 2018.

What Leonard described earlier about having opportunity to play together with others, and Connie’s reflections on Paul’s experiences matter, because such occasions can build feelings for individuals of positive emotions and wellbeing (see, also, Brajša-Žganec et al., 2011). Further, involvement in this way alongside peers can contribute by supporting resilience. Such examples resonate with findings of Morgan et al., (2013), who reported on ‘team resilience’, which in turn play a part in the conditions for the creation of safe spaces. The value of Centre spaces in providing this powerful feeling of communality, with their inherent diversity of shape, design and size, was articulated by Jacqui, to a degree, when she reflected on the ways she had engaged with the space and referred to the various environments available for use at her setting:

“…it’s nice that there’s the facility for us all to be in our various ways”.

Jacqui: interview at her Centre, 4th October 2018.

Time spent within Centre spaces appears notable in helping individuals express meaning about their participant identity and place in the world. What individuals brought with them in terms of life story mattered, as did the opportunity spaces afforded for relationship crafting. Here, it is useful to clarify the learning that accrued for us as researchers from these experiences so that insights might inform practice. This is challenging in the context of the present discussion, informed by the experiences of different individuals, places where the diverse nature of spaces on offer has been shown to be significant, and the comprehensive scholarship drawn from the fields of dementia and leisure. It is to the theory of the opportunity structures that we now turn.

#### Opportunity Structures

Opportunity structures, as configured in this research, are the theoretical understanding whereby disabled participants use leisure and physical activity to reframe a crisis of identity to shape an ongoing sense of self (Lundberg et al., 2011). Lundberg and colleagues argued that sporting activities could provide a context through which a stigmatized identity could be redefined. In our study opportunity structures operated on identity of participants within the spaces in three ways, which sometimes overlapped. This was through the physical activity itself; via opportunity structures that helped people adjust to living with dementia; and the diversity of activity as opportunity structures at play within Centres.

First, the physical activity itself enabled opportunity structures to function either through how participants felt about their engagement with activities, or from feedback received from others following engagement. Feelings of freedom reported by participants are illustrative and helpful in this regard. For example, Ivan’s freedom was drawn from the personal and communal. He utilised personal encounters through leisure participation at the Centre to find out more about himself. However, he did not appear to be an individualist. It was not exclusive personal freedom he was seemingly seeking. Rather, as in harmony with Anderson and Whitfield (2013), being part of a social group mattered because it provided a source of positive emotions, a sense of belonging, and purpose amid the threats posed by illness. This resonated with Ivan’s earliest recollections of his time and feelings about playing sport with his schoolfriends:

“…it was a bunch of people…to play with…because there was always a lot…you come during the holidays and you never went without something…so they would come from school…so brilliant.”

Ivan: interview at home, 12th December 2017.

Ivan’s wife Jemma added:

“He would come in eat his dinner, and go out and play football again with his friends across the park… I wish some of his friends were here because they would tell you some of the tales.”

Jemma: interview at home, 12th December 2017.

This sense of freedom at the end of every school day or in the holidays could be replicated as freedom from dementia within the communal spaces afforded by the Centre. Ivan was aware of the restrictions the symptoms of the corticobasal degeneration placed upon him. For example, in terms of his impaired speech and manual dexterity. How he felt about this and the response of others engaging in leisure at the Centre alongside him is illustrated by the following observation:

“At one point another member of the class subtly and sensitively handed Ivan back his weight when he dropped it. She used his name. Ivan clearly appreciated the gesture and, I think, how it was done. In other words discreetly, without fuss…”

Ivan: observation at his Centre, 23rd March 2018.

Freedom can be portrayed as a blend of being an individual without constraint, but within the ‘safety net’ of community (Cohen-Gewerc & Stebbins, 2013, pp. 6–7). Here was an environment where Ivan could be free in the sense he could utilise leisure activities to be who he genuinely wanted to be, whilst recognising he was operating within an environment where communal support was at hand if he required it. The fact this community was based upon the leisure he loved meant those activities could act as opportunity structures, either through how he felt about his own participation, or in the manner demonstrated in the preceding example, through feedback offered by others. This matters because freedom of choice in activity is essential to realising freedom for people with dementia in the leisure context (Noone & Jenkins, 2018). We appreciate that Ivan’s experience may be unique to his setting, situation and the conditions provided by his specific Centre. However, what is insightful about this is that Ivan’s example highlights how the safe spaces we discussed earlier can be manifested in ways that feel positive and progressive to the participant. The activity was an opportunity structure which enabled Ivan to continue to shape his identity in a way where he felt free, all the while within a space where he felt safe.

The second way in which opportunity structures operated was in the manner in which they helped people adjust to living with dementia. To do so the offering was tailored to support people to be active citizens, in the manner suggested by Birt et al., (2017) to sustain participatory roles within daily life. This was shown by the example quoted earlier where Jacqui reflected:

“…it’s nice that there’s the facility for us all to be in our various ways”.

Jacqui: interview at her Centre, 4th October 2018.

Opportunity for adjustment is also dependent, however, upon what is offered being organised so it enables people to recognise how engagement is having an impact upon how they perceive themselves. Lundberg et al., (2011) summarised this as individuals being enabled to note feedback relating to identity and use it to shape their ongoing sense of self. Jacqui summarised this when she reflected on what participation in physical activity meant for herself:

“The more you can still continue to do for yourself … not necessarily holding it at bay, but keeping a par with it (the Dementia)… There’s a life out there.”

Jacqui: interview at her Centre, 4th October 2018.

Finally, diversity of activity operated as opportunity structures within Centres. Centres can help people express continuity of identity through the flexible and diverse nature of activities they make available. This offers participants opportunity for choice, aspiration, agency, and authenticity. Each of which and in combination can strengthen identity and social citizenship. Performativity, performing a role through engaging in physical activity (Spracklen, 2013), enables a person living with dementia to sustain their presence in the social world (Genoe, 2010). Centres offer opportunities for such performativity. For example, Leonard was able to make choices about what he did at the Centre, and the reasons he elected to do so, as in the earlier example about his choice of badminton over table-tennis, because of the former offering opportunity for the sort of vigorous leisure he preferred (see page 22, above). Additionally, as he said about what he valued in relation to the options his Centre offered him for physical activity:

“It’s the variety, there’s lots of things to do… I can do lots.”

Leonard: go-along interview at his Centre, 22nd January 2018.

Paul was able to choose activities as diverse as dominoes and table tennis. This was appreciated by him too, as Connie reflected:

“…that’s what we do, we take dominoes and play a game… there again, he likes to win, you know!”

Connie: interview at home on 14th March 2018.

## Discussion

Centres are spaces where people living with dementia can act in ways which challenge the paradigm that people living with the condition are necessarily vulnerable (Bartlett & Brannelly, 2019). We have illustrated where Centres were used by participants as spaces to ameliorate the harm and adversities traditionally associated with dementia, for instance, related to changing symptoms and pathologies, and social isolation and stigma concerns. ‘Opportunity structures’ were used by participants and Centre Workers to enable transitions, whereby participants moved beyond societal perceptions of individuals living with dementia as fragile vessels, exclusively in need of protection, to understandings of people with full membership of society. Essentially, to be seen as people who continued to contribute, often in partnership with others, and who were able to prosper (Swann, 1987; Swann and Bosson, 2008; Sharp, 2019). Use of Centre spaces by people living with dementia enabled sustenance of identity and the strengthening of feelings of ‘being in the world’.

Accordingly, we have advanced the value of Centres as opportunity structures, reflecting wider arguments related to social networks and care structures in public health debates (Cornwell & Laumann, 2015; Ellward Van Tilburg and Aartsen, 2015; Fratiglioni et al., 2000; Huxhold et al., 2013; Wiersma & Denton, 2016). In examining Centres’ roles more closely, our work here contributes to collective encouragement of practitioners, leaders and policy makers to note the value opportunity structures offer in relation to identity formation and the ability of people living with dementia to sustain their social citizenship. Through our work we call for greater attention to the ways physical activity is designed and offered, maintaining a focus on how activities can support people adjusting to living with dementia and providing a diversity of activity. The critical interdisciplinary approach we have taken in interrogating opportunity structures at the nexus of leisure, sport and dementia studies is, moreover, helpful in making sense of a context where it remains essential to recognise the diversity of each individual with dementia (Kitwood, 1997), and where the spaces in question will themselves be diverse in nature and shape.

Ultimately, Centres offer people living with dementia occasion to be agentic in what they do and how they go about it. Notions of agency in participant accounts, however, also appeared enmeshed with issues of authenticity and (re)presentation. This is not, we understand, unique to people living with dementia. Rather, as Spracklen (2013) suggests, a common occurrence with most peoples’ engagements with leisure activity. Yet, within our research, authenticity-of-self held additional piquancy for understanding the ways participants’ sense of identity and belonging had been destabilised by their condition, and also how Centres could provide spaces for (re)building a sense of one’s self and ascribing it meanings and purposes that validated participants’ presence in the world. Nonetheless, we acknowledge too that the findings raise questions regarding how authenticity is conceived, and potentially capitalised on to enhance Centre spaces, that warrant further exploration.

Connections between physical, social, economic and political aspects of space were evident also with regard to ideas of care and welfare provision, and the potential framing of Centres as ‘safe’ spaces. In witnessing the ability of Centres to become sites of security and sanctuary for participants amid the challenges encountered in their daily lives, our research led us to these wider contemplations about the conceptualisation of Centres. Specifically, as acknowledging Centres’ sites within which the harm traditionally associated with dementia could be ameliorated. In advocating for the creation of safe leisure spaces, we acknowledge that dialogue and change is already being undertaken across the sport, recreation and physical activity sector. Work here has, for instance, highlighted efforts being made by leisure providers to exhibit better sensitivity and sensibilities towards constituents and particular population demographics, for example, youth, older populations, HIV/AIDs, LGBTQ (Anderson et al., 2018; Oosthuizen and Burnett, 2019; Jones et al., 2021). These directions, in tandem with our research, provides encouragement for finding ways for Centres to be fostered as ‘safe spaces’ by capitalising on their possible links to care and support through sustaining wellbeing, participation and meaningful engagements. This might apply, we appreciate, not just for people living with dementia, but for a much wider community of sport/leisure participants.

The insights provided here are timely because of the relationship between adequate social, economic and political support/resourcing and the ability of Centres to facilitate and sustain meaningful and safe spaces. The consideration of Centres in this light has, we note, become particularly pronounced during the Covid-19 pandemic and ongoing debates over its consequences and legacies on public health spaces and services (BBC, 2021; Horton, 2020). More broadly there is relevance in relation to organisational duties of care, both legal and moral. These issues are evermore paramount given the prevailing context of health and social care in the UK (and beyond), the continued austerity and struggles faced in some communities (BBC, 2021). There is value, therefore, in extending our arguments to wider contexts, and other places. For example, to social care provision and addressing individual care needs within communities.

## Conclusion

This research built upon recognition among scholars that social networks forged at the community level constitute a significant asset in the care structures that comprise dementia resourcing (Odzakovic, 2021; Wiersma & Denton, 2016; Wright, 2018). Learning from our study might be extended to wider contexts. For example, through including Centres within formalised social care and health initiatives. This also connects with broader global issues in particular relating to health, social isolation, and aging populations (Wu, 2020), and to salutonogenic paradigms of health, whereby individuals actively engage with their health through leisure (Peel et al., 2021). In turn there might be scope to include social prescription within the context of participation of people living with dementia within Centre settings, as highlighted earlier. Whilst detailed consideration of the practicalities is beyond this paper’s scope, careful thought must be given before initiatives such as those described above are incorporated within formalised schemes. This is because, as Spracklen (2013) contends, leisure carries with it transformative potential in being a communicative space within which individuals can create new meaning.

Whether organised and/or ‘prescriptive’ offerings in Centres can be transformative may, we accept, be remain debatable. Nevertheless, what is exciting and constructive about the possibility of including leisure within more organised approaches is the scope that this affords for people to participate as citizens instead as patients or those to be ‘cared for’. This shift is fundamental because it helps to counter any tendency to ‘other’ people living with Dementia that is rife within formal services, and indeed wider society, and is responsible for so much of the paternalism (conscious and unconscious) that strips away the personhood of people unnecessarily and to their detriment (Fletcher, 2021). Even so the caution alluded to above must be maintained. What Centres can provide will not be for everyone, because, for example, some will not want nor physically be able to engage with activity. Also,—and as has become so profound during the pandemic—Centres are limited by their own resources and priorities. Additionally, efforts toward enabling and sustaining social citizenship might fail. Yet, it appears that Centres with a not-for-profit ethos and value base focused upon community benefit were well-placed for participants in addressing such uncertainties, ameliorating harm, and enabling individuals to prosper.

## Data Availability

The datasets generated during and/or analysed during the current study are not publicly available because they primarily relate to the circumstances of four people living with dementia and their families but are available from the corresponding author on reasonable request.
